# The Biological, Histopathological, and Parasitic Abundance of *Oreochromis niloticus* Inhabiting Two Different Freshwater Canals

**DOI:** 10.1007/s00128-022-03639-8

**Published:** 2022-12-22

**Authors:** Marwa I. Saad El-Din, Mahi A. Ghobashy, Farida A. Mansour, Nahla S. El-Shenawy, Heba N. Gad EL-Hak

**Affiliations:** Zoology Department, Faculty of Science, Suez Canal University, Ismailia, 41522 Egypt

**Keywords:** Liver, Gonads, Parasites, Metals, Fish

## Abstract

The current study aimed to assess the impact of metal pollution in water on parasitic abundance, biology, and histopathological characteristics of *Oreochromis niloticus* in two different freshwater habitats in the Al Sharkia government. The fish were collected from the Mueweis canal area (A) which received industrial wastewater from factories and the San El-Hagar area (B) which received agricultural and domestic wastewater. Parasitic abundance and histopathological changes in the liver and gonads were inspected. The total prevalence of parasitic infection was at the highest percentage in area B correlated with metals present in the water, in addition to severe histopathological damage to the liver and the gonads. The prevalence of parasites for different examined fish ranges from 50% for parasites in the San El-Hagar canal and 4.17% for parasites in the Muwies canal, seasonally. There were positive relationships between Fe or Zn or Mn concentrations with parasite abundance in tilapia fish collected from the San Hagar canal. Several histopathological alterations were detected in the liver and gonads of *O. niloticus* collected from the two canals located in the Al Sharkia province. It was concluded that the uncontrolled inputs of agricultural and domestic wastes highly altered the *O. niloticus* health status and the prevalence of the parasites in the investigated two areas.

Fish responses to environmental stressors can be correlated with their anatomical positioning, route of exposure, and distribution pattern of contaminants, in addition to defense ability (Stoliar and Lushchak 2012). They contribute to about 6% of the world’s supply of protein and about 24% of animal protein (Béné et al. 2015). They are the most abundant and significant source of protein for low-income populations, in tropical and subtropical countries and it is highly consumed by a large sector of Egyptians compared to other Egyptian fish (Deng 2020). They are inhabiting fresh water and water bodies of low salinity (Melo et al. 2019). Ben Ameur et al. (2012) suggested that fish play a two-fold function of being on the uppermost level of the food chain and reacting powerfully to environmental stress (Al-Halani 2018). *Oreochromis niloticus* is one of the ideal models for the evaluation of bioindicators of pollution (Zhou et al. 2008).

In fisheries biology, the length-weight relationship (LWR) of fishes is essential because it is used to estimate the weight associated with a particular length (Sarkar et al. 2013). In addition, LWR provides data on fish conditions and growth trends (Ighwela et al. 2011). Condition factor has been utilized as an indication of health (Morton and Routledge 2006) that decrease with an increase in length (Jisr et al. 2018).

Fish is prone to diseases like parasitism and can also be a good host for parasite multiplication (Davies and Johnston 2000). Parasites can be acquired by humans through the ingestion of raw or inadequately cooked fish (Teklemariam et al. 2015). Fish quality is an important problem in the world of fish eating (Raatz et al. 2013). Essentially, the goal of the fish evaluation is to prevent consuming contaminated food and to examine the nutritional content of food to assure the consumer’s safety (Teklemariam et al. 2015). The usage of pathogenic fish has been linked to major health problems in humans (Rahman et al. 2020).

*O. niloticus* is highly demanded as food in the selected investigated areas. Moreover, the study of *O. niloticu’s* health in these areas is limited. The objective of the present study is to provide a recommendation(s) for the governmental, environmental, and economic sectors in the context of working in biomonitoring this area from the industrial, domestic, and agricultural waste which affects the fish health state and may also affect the human health.

The present study aimed at evaluating the biological characteristics and parasitic abundance of *O. niloticus* in two different polluted freshwater habitats and provide information regarding histopathology of the liver and gonads to determine whether the fishes are in good condition.

## Materials and Methods

**Fig. 1:**
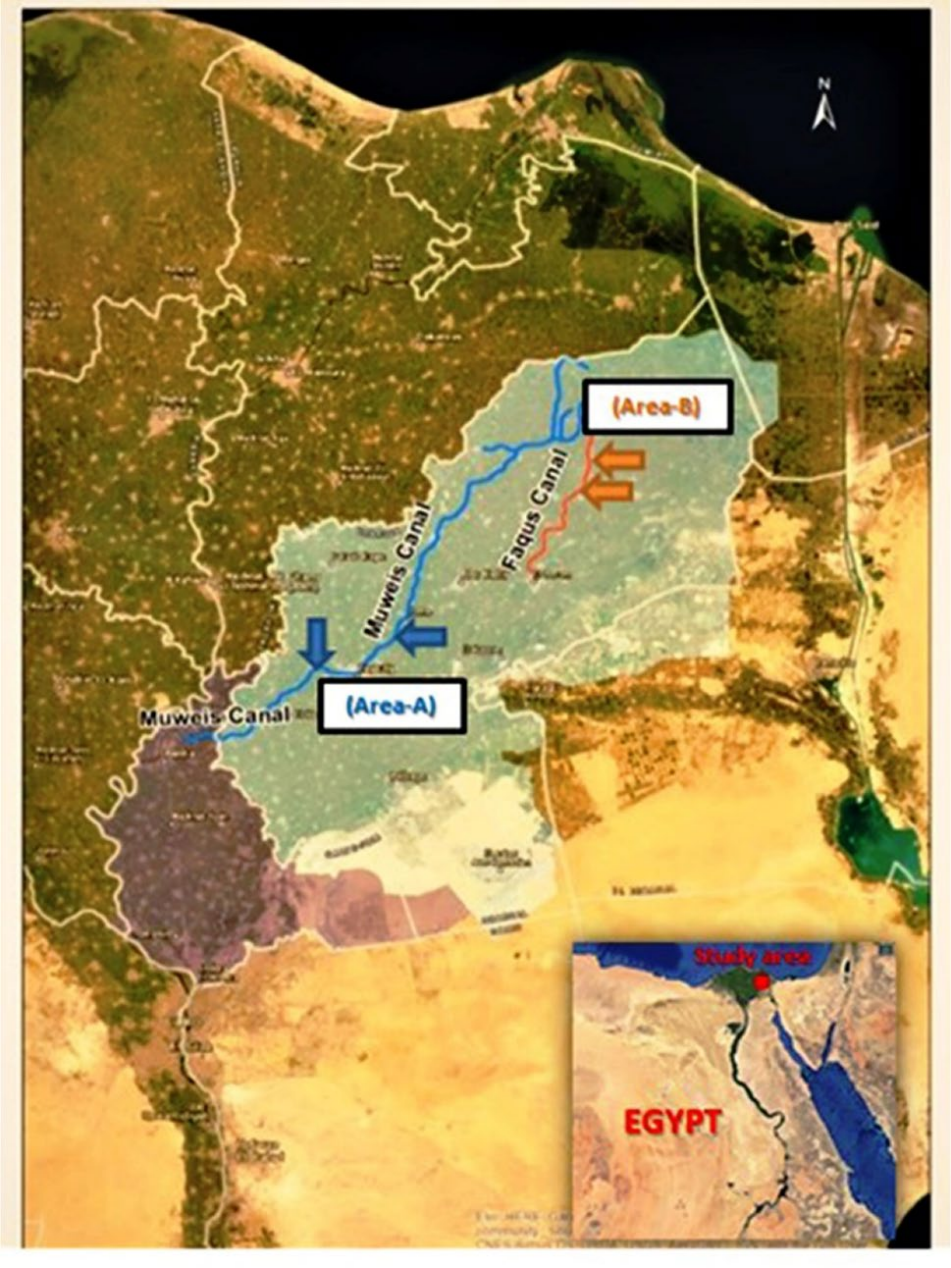
Map of the sampling areas of Muwies and San El-Hagar canals located in the Al Sharkia province

Samples (water and fish; n = 100) were collected from two localities in Al-Sharkia Province which are considered natural and famous locations for fishing. Muweis canal lies at the center of Zagazig city (30° 35′ 34″ N and 31° 25′ 46″ E) (at ADab Bridge which receives industry pollution from factories) and San El-Hagar canal lies north of Faqus city (30° 96′ 50″ N and 30° 76′ 85″ E) (at south San El-Hagar Bridge which receives agricultural and domestic pollution). Samples were collected monthly from the two localities from September 2017 to August 2018 (Fig. 1). The physical and chemical properties of the water in the collection areas are previously mentioned in Mansour et al. (2019) and El-Hak et al. (2022).

A total number of 192 adult fish (96 fish/site) of *Oreochromis niloticus* with an average body length of 21.24 cm ± 0.20 and average body weight of 170.05 g ± 5.16 were gathered with the assistance of fishermen from Muwies and San El-Hagar canals, respectively. Then Live fish were transferred to the laboratory in polyethylene bags filled with air and water, where they were kept in aerated glass aquaria until they were evaluated for the following analysis within a maximum of 1 day.

Using a meter rule, the total length (TL, cm) of each fish was measured from the tip of the closed mouth to the expanded tip of the caudal fin. The TL was then measured in millimeters for each fish using a measuring board. A computerized top-loading electronic weighing balance was used to determine the body weight (BW, g) (Fafioye and Oluajo 2005).

The data on the growth pattern of the species were collected through the length (L) and weight (W) relationships. The statistical link between these fish parameters was observed by using the parabolic equation which transformed into logarithmic form to give a regression equation and a straight line as follows:

$${\text{W }} = {\text{ a L}}^{{\text{b}}}$$

$${\text{Log W }} = {\text{ log a }} + {\text{ b log L}}$$

where W is the weight of the fish in grams (g), L denotes the total length of the fish in centimeters (cm), and a denotes the exponent characterizing the rate of change of weight with length (= the intercept of the regression line on the Y-axis), and b denotes the slope of the regression line (the Allometric coefficient). The determination coefficient (r²) was used to calculate the degree of relationship between TL and TW. When b = 3, the fish develops symmetrically or isometrically (assuming its specific gravity remains constant); otherwise, it is positive allometric if b > 3 and negative allometric if b 3. The exponent (a) is found to range between 2.5 and 4.0. If that exponent is 3.0 or close to it, growth is said to be isometric.

Fish with thin, elongated bodies have b values less than 3, whereas fish with broader bodies have b values more than 3. When b = 3, isometric growth occurs, and little specimens experience the same conditions as giant ones. Negative allometric growth or tiny specimens are in better condition than large specimens when b 3. When b > 3, or when large specimens are in better condition than small specimens, they grow in height or breadth quicker than in length, either as a consequence of a change in body form with size or because the large specimens in the sample are in better condition than the little ones.

The condition factor “K” describes the condition of the fish based on the following relationship according to Gupta et al. (2011).

$${\text{K }} = \left( {{\text{TW }}/{\text{ TL}}^{{\text{3}}} } \right)\times 100$$

where K = condition factor, TW = total weight of fish (g), TL = total length of fish (cm), and 100 is a factor to bring the value of K near the unit. It is used to compare the condition, fatness, or well-being of fish, with the notion that heavier fish of a particular length are in better shape. As a result, fish with a condition factor of more than one (1) were labeled high, while those with a condition factor of less than one (1) were classified as low. Fish in good shape will have greater K levels than those in bad shape.

The fish *O. niloticus’* liver and gonads were carefully removed and promptly fixed in Bouin fixative for 48 h before being dehydrated in escalating grades of alcohol and cleaned in xylene. The preserved tissues were immersed in paraffin wax and sliced into 4–6 microns using a Euromex Holland microtome. The Harris hematoxylin and eosin (H&E) procedure was used to stain the sections (El-Hak et al. 2021). They were then examined under a microscope and photos were taken by a microscope camera.

*O. niloticus* organs were separated and put on Petri plates containing saline solution. Each organ’s outside surface was examined, and then the organ was opened to search for parasites. A stereomicroscope and a compound microscope were used to visually evaluate the presence of parasites in each fish organ and cavity. The prevalence (average number of infected fish per examined fish), the intensity of infection (average number of parasites per infected fish), and the abundance (average number of parasites per examined fish) were calculated according to Rózsa et al. (2000).

Three water samples were collected seasonally from the two locations. The water samples were mixed and acidified with pure HNO_3_. The concentration of total metals in the water samples was analyzed within 24 h and expressed as mg/L. Determination of metals concentrations (Fe, Zn, Mn, Cd, Pb, Ni, and Cu) in the digested water samples was done by comparing absorbance with known certificated standards for the selected metal using an Acetylene Flame Atomic Absorption Spectrophotometer, AES 2000 series according to Shar et al. (2013). The water samples were digested by adding 10 mL of ultrapure concentrated HNO_3_ and left overnight, then they were heated on a hot plate at 70°C for 8 h. After the solutions cooled, 4 mL of H_2_O_2_ (30%) was added and heated again at 70°C for 4 h. The solution was diluted with deionized water to 25 mL. Every digestion contained a blank and two verified materials for quality control. Atomic absorption spectrophotometer (Varian AA-7000) measurements of metals were performed using external calibration. For instrument standardization, a standard stock solution of 1000 mg/L multi-element (Merck, Germany) was utilized. Every sample was examined three times, and if the variation coefficients were less than 5%, they were determined and accepted. The quality control samples’ analytical results for metal determination performed satisfactorily within the 95%–100% approved values range. According to the results of the blank and drift standards, no drift was found during the examination. To prevent metal contamination, glassware was first immersed in 10% nitric acid and then rinsed with deionized water before use. All reagents were of analytical quality.

Statistical regression analysis was determined to estimate the relationship between the abundance of parasites and the body weight of fish as well as the correlation of prevalence of parasitic infections, parasitic load (%), and condition factor (K) with the fish length. Data presented as mean ± S.E. and P value less than equal to 0.05 using Two-way ANOVA of variance. Pearson rank correlation was made to determine if there was a relationship between metal levels and parasite abundance. All statistical tests were considered statistically significantly different when *p* ≤ 0.05.

## Result

**Fig. 2:**
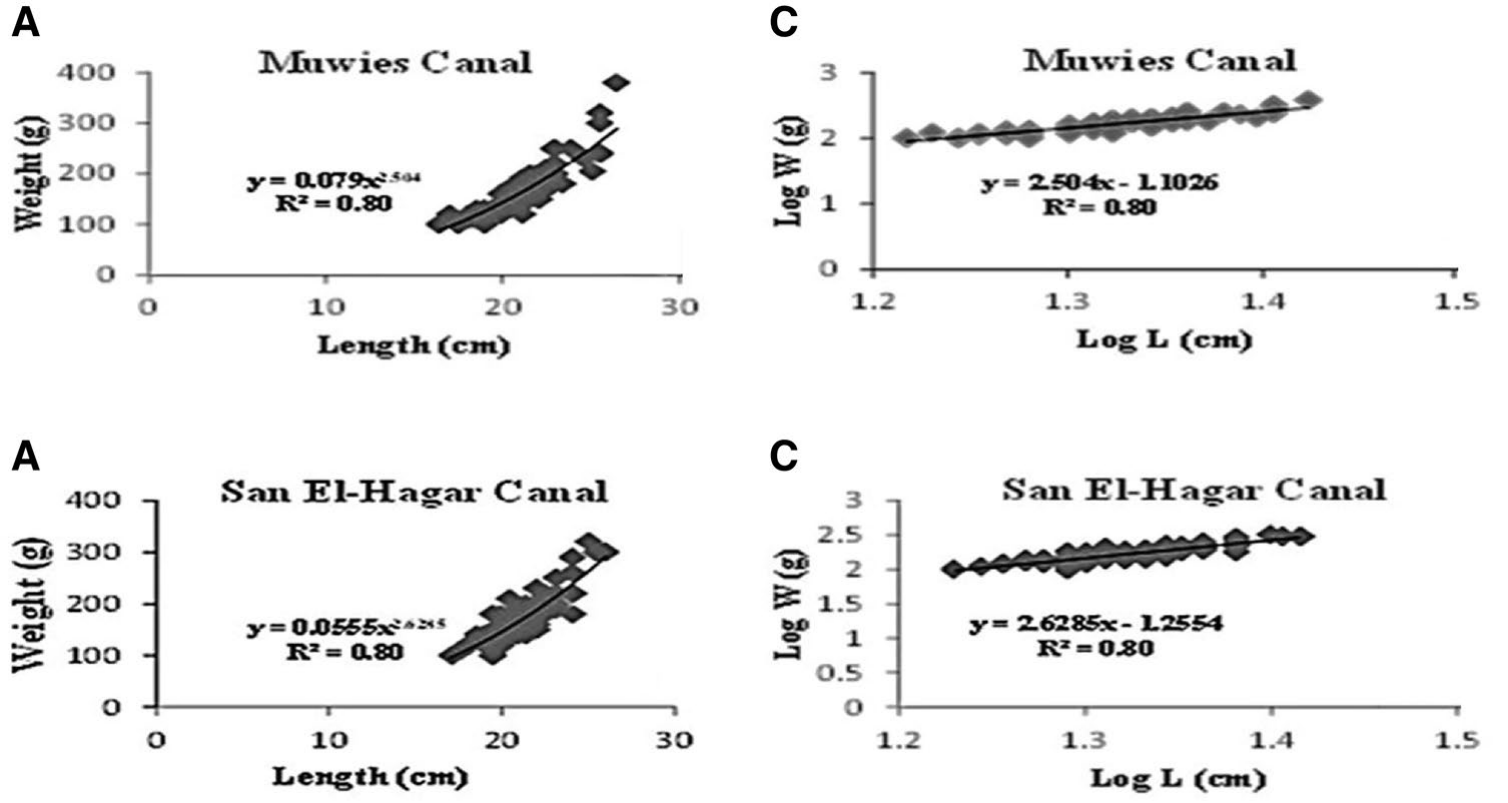
The Length-weight relationship and the Log-log relationship of *O. niloticus* from Muwies and San El-Hagar canals. **A** and **B** showed the length-weight relationship of *O. niloticus* from the Muwies and San El-Hagger canals, respectively. **C** and **D** showed the Log-log relationship of *O. niloticus* from the Muwies and San El-Hagar canals, respectively

Only the length-weight association equations for both sexes were established (Fig. 2). 192 Adult fishes (96 fish/site) were examined during this study from the two polluted canals. The data were pooled irrespective of sex and one relationship between fish total length and total weight in Muwies canal and San El-Hagar canal was derived, respectively as:

$${\text{Log W }} = - {\text{1}}.{\text{1}}0{\text{26 }} + {\text{ 2}}.{\text{5}}0{\text{4 L or TW}} = 0.0{\text{79 }}*{\text{ L}}^{{{\text{2}}.{\text{5}}0{\text{4}}}} \left( {{\text{Muwies Canal}}} \right)$$

$${\text{Log W }} = - {\text{1}}.{\text{2554}} + {\text{ 2}}.{\text{6285 L or TW}} = 0.0{\text{555 }}*{\text{ L}}^{{{\text{2}}.{\text{6285}}}} \left( {{\text{San El-Hagar Canal}}} \right).$$

**Table 1:** The seasonally length–weight relationship, regression equation, regression coefficient (R^2^), and condition factor of the collected *O. niloticus* (n = 100)

Season	Muwies Canal							
	Length (cm)		Weight (g)		Regression equation		Regression coefficient	K-factor Mean ± S.E
	Range	Mean ± S.E	Range	Mean ± S.E	a	b	R^2^	
Summer	18.5–23	20.71 ± 0.31	120–250	157.92 ± 9.17	0.04	2.71	0.71	1.76 ± 0.05
Autumn	20.5–22.5	21.46 ± 0.17	160–210	179.38 ± 3.96	0.53	2.08	0.58	1.81 ± 0.03
Winter	16.5–26.5	20.88 ± 0.77	100–380	179.17 ± 19.47	0.50	2.68	0.95	1.89 ± 0.05
Spring	19–25.5	21.94 ± 0.45	100–240	163.75 ± 8.98	0.07	2.49	0.83	1.55 ± 0.04

Season	San El-Hagar Canal							
	Length (cm)		Weight (g)		Regression equation		Regression coefficient	K-factor Mean ± S.E
	Range	Mean ± S.E	Range	Mean ± S.E	a	b	R^2^	
Summer	19–23	20.67 ± 0.32	110–250	159.46 ± 8.52	0.01	3.08	0.78	1.79 ± 0.04
Autumn	17–25	19.79 ± 0.44	100–320	160.42 ± 12.15	0.03	2.90	0.86	2.03 ± 0.05
Winter	19–26	22.75 ± 0.49	120–300	212.08 ± 14.59	0.01	3.29	0.92	1.76 ± 0.04
Spring	18–24	20.80 ± 0.41	100–200	149.38 ± 7.60	0.21	2.17	0.68	1.66 ± 0.06

The ranges and averages of the total length and body weight of O. niloticus are shown in Table 1, as well as the length-weight connection (a and b parameters), condition factor, regression coefficient (R^2^), and regression equation. The total length of *O. niloticus* fish samples, collected from the Muwies canal varied between (16.5–26.5 cm) whereas the total body weight varied between (100–380 g). On the other hand, the total length of *O. niloticus* samples, collected from the San El-Hagar canal varied between (17–26 cm) and the total weight varied between (100–320 g). Fish collected from the San El-Hagar canal had a higher mean total length (22.75 cm) during winter, a larger mean body weight (212.08), and a smaller mean condition factor (K).

As shown in Table 2, reveal 44 infected fish out of 192 fish examined in the two studied areas of Al Sharkia Governorate. Overall prevalence was 22.92%, with a mean intensity of 4.09 and an abundance of 0.94. The general prevalence of parasites was 35.42% and 10.42% in San El-Hagar and Muwies canals, respectively. It was concluded that the highest rate of parasite infection was observed during the spring season, with infection rates of 50% in the San El-Hagar canal, followed by winter (45.83%), then autumn was recorded at 41.67% and summer showed the lowest infection in the examined fish (4.17%) while in Muwies canal, parasite infection was recorded only in winter with an infection rate of 41.67%. The highest intensity rate of the parasite was recorded in winter at San El-Hagar (5.09), followed by autumn (5.0), spring (3.67), and summer season (3.0). The lowest rate of intensity of the parasite was found in the Muwies canal during winter (2.7).

**Table 2:** Prevalence (P%), mean abundance (MA) of parasites, mean intensity (MI) in *O. niloticus* (n = 100) inhabiting different habitats

Seasons	Areas	EF	IF	N	P%	MA	MI
Summer	Muweis	24	–	–	–	–	–
	San El-Hagar	24	1	3	4.17	0.13	3
Autumn	Muweis	24	–	–	–	–	–
	San El-Hagar	24	10	50	41.67	2.08	5
Winter	Muweis	24	10	27	41.67	1.13	2.7
	San El-Hagar	24	11	56	45.83	2.33	5.09
Spring	Muweis	24	–	–	–	–	–
	San El-Hagar	24	12	44	50	1.83	3.67
Total		192	44	180	22.92	0.94	4.09

N; the number of collected parasites, EF; the number of examined fish, and IF; the number of infected fish.Variations of condition factor (K) with total length (TL)

**Table 3:** Pearson coefficient correlation between the metal concentration of water in the two studied areas and the parasite abundance

Muwies canal		San Hagar canal	
Metal of water (mg/L)	Parasite abundance	Metal of water (mg/L)	Parasite abundance
Fe	− 0.158	Fe	0.72*
Zn	− 0.207	Zn	0.753*
Mn	− 0.383	Mn	0.705*
Cd	− 0.109	Cd	–
Pb	− 0.088	Pb	–
Cu	− 0.351	Cu	–

(*) is a significant difference *P* ≤ 0.05. (–) below the detected limit

Pearson coefficient correlation between the metal concentration of water in the two studied areas and the parasite abundance in tilapia fish (Table 3). No relationships were found between metal concentrations of water in the Muwies canal and with parasite abundance of tilapia fish collected from that area. In addition, there were positive relationships were found between Fe or Zn, or Mn concentrations with parasite abundance in tilapia fish collected from the San Hagar canal. As the Fe, Zn, and Mn concentrations increase in the water the parasite abundance increase. Thus, the parasite abundance appeared to be primarily dependent on metal concentrations in the water of the San Hagar area.

**Fig. 3:**
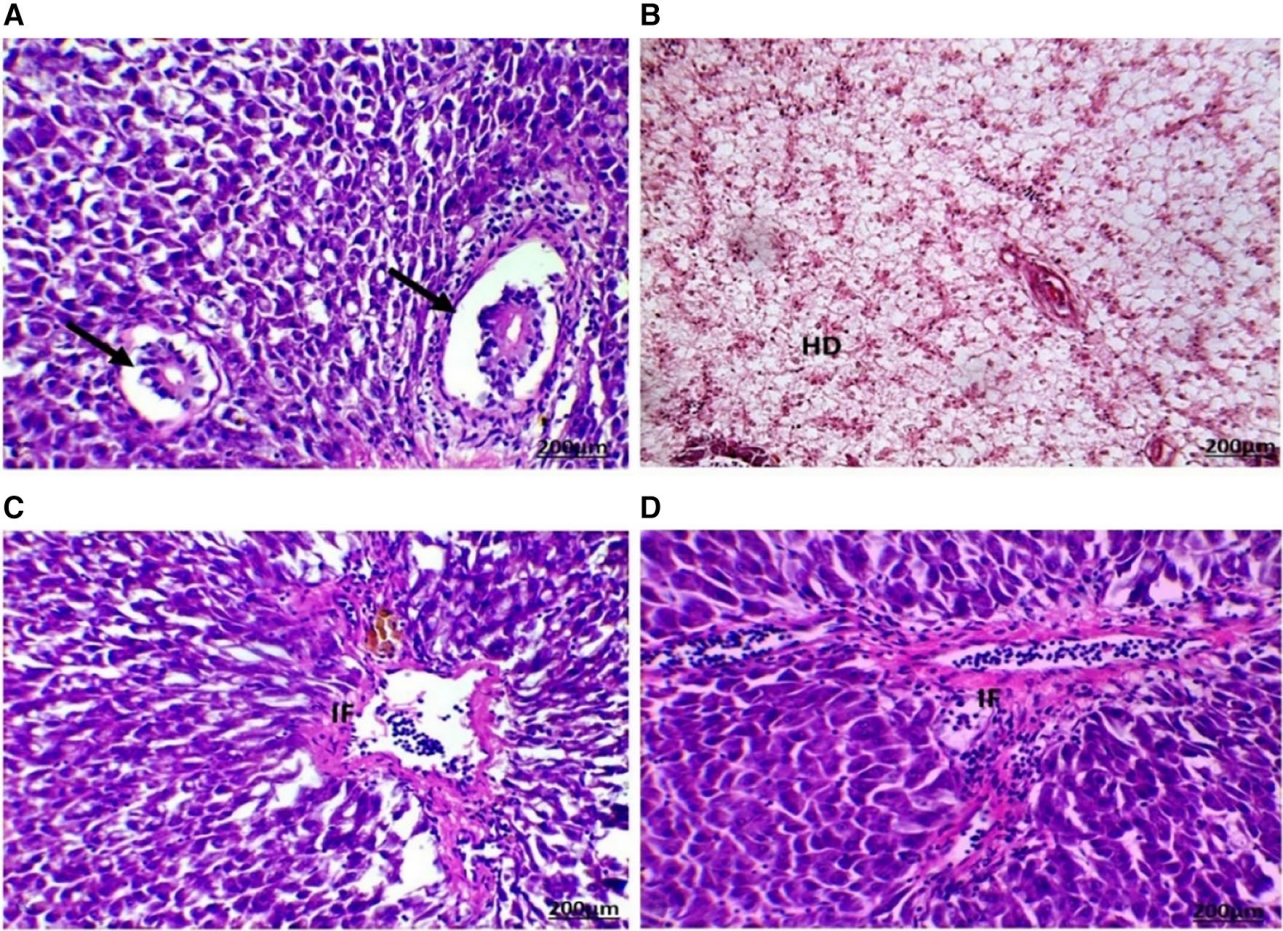
The liver of fish in area A during summer (**A**) showed infiltration of inflammatory cells surrounding the hypertrophy bile duct (arrow). Autumn (**B**) hydrobic degeneration (HD) in the central and portal area. Spring (**C**) and winter (**D**) seasons showed infiltration of inflammatory cells (IF) between the hypertrophic hepatocytes (HE, 200X)

**Fig. 4:**
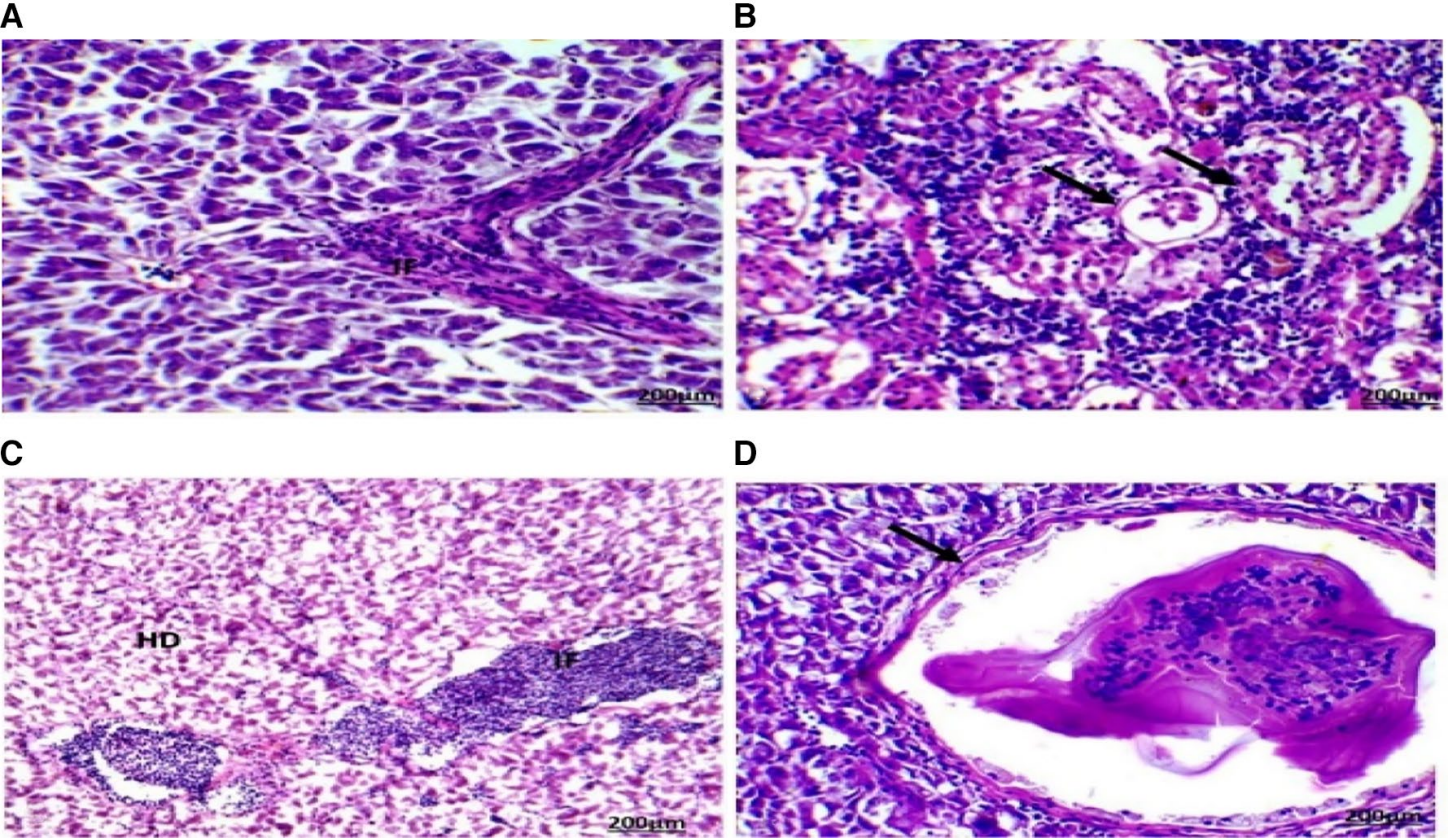
The liver of fish in area B during summer (**A**) showed infiltration of inflammatory cells (IF) between the hepatocytes. Autumn (**B**) showed aggregations of inflammatory cells (IF) between the hepatocytes with the destruction of hepatocytes arrangement response to the encysted metacercaria. Spring (**C**) showed aggregations of inflammatory cells (IF) between the hepatocytes. Winter (**D**) showed aggregations of inflammatory cells surrounding the encysted metacercarial cysts (arrow) with irregular double-layer walls and folded bodies (HE, 200×)

Several histopathological alterations were detected in the liver and gonads (testes and ovaries) of *O. niloticus* collected from the two canals located in the Al Sharkia province (Figs. 3 and 4).

The highest number and severity of different histological alterations were identified in fish collected from San El-Hagar during the four seasons. Congestion in the portal area accompanied by infiltration of inflammatory cells was a predominant feature in fish livers from the two sampling sites and was identified in fish from Muweis and San El-Hagar during the four seasons. Numerous histopathological changes were found to be extensive in fish during all seasons. Infiltrations of inflammatory cells especially with a response to encysted metacercaria were the most prevalent in livers compared to any of the other types of alterations. Hydropic degenerations were identified in fish from Muweis and San El-Hagar canals during the four seasons.

**Fig. 5:**
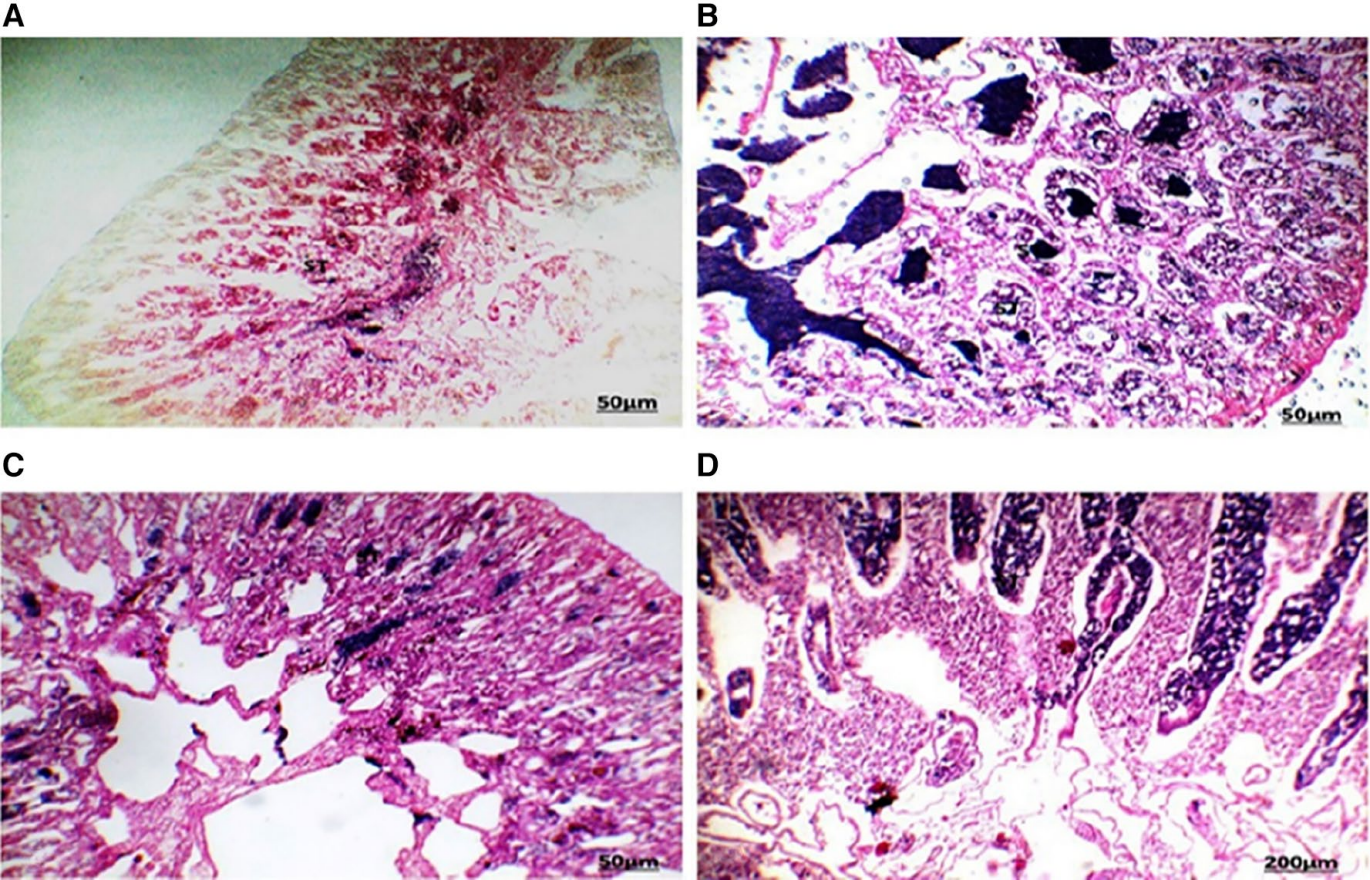
Section of testis collected from area A during summer (**A**) showed small testicular lobules (ST) with thin tunica albuginea and only spermatogonia and appeared kidney in shape. Autumn (**B**) showed some testicular lobules (ST) with dense spermatozoa in the lumen and degenerated spermatogenesis. During spring (**C**) and winter (**D**) seasons showed some empty degenerated testicular lobules with all the developmental spermatogenic stages and thick tunica albuginea, the seminiferous tubules take the shape of parachute and are broken (HE, 100×)

**Fig. 6:**
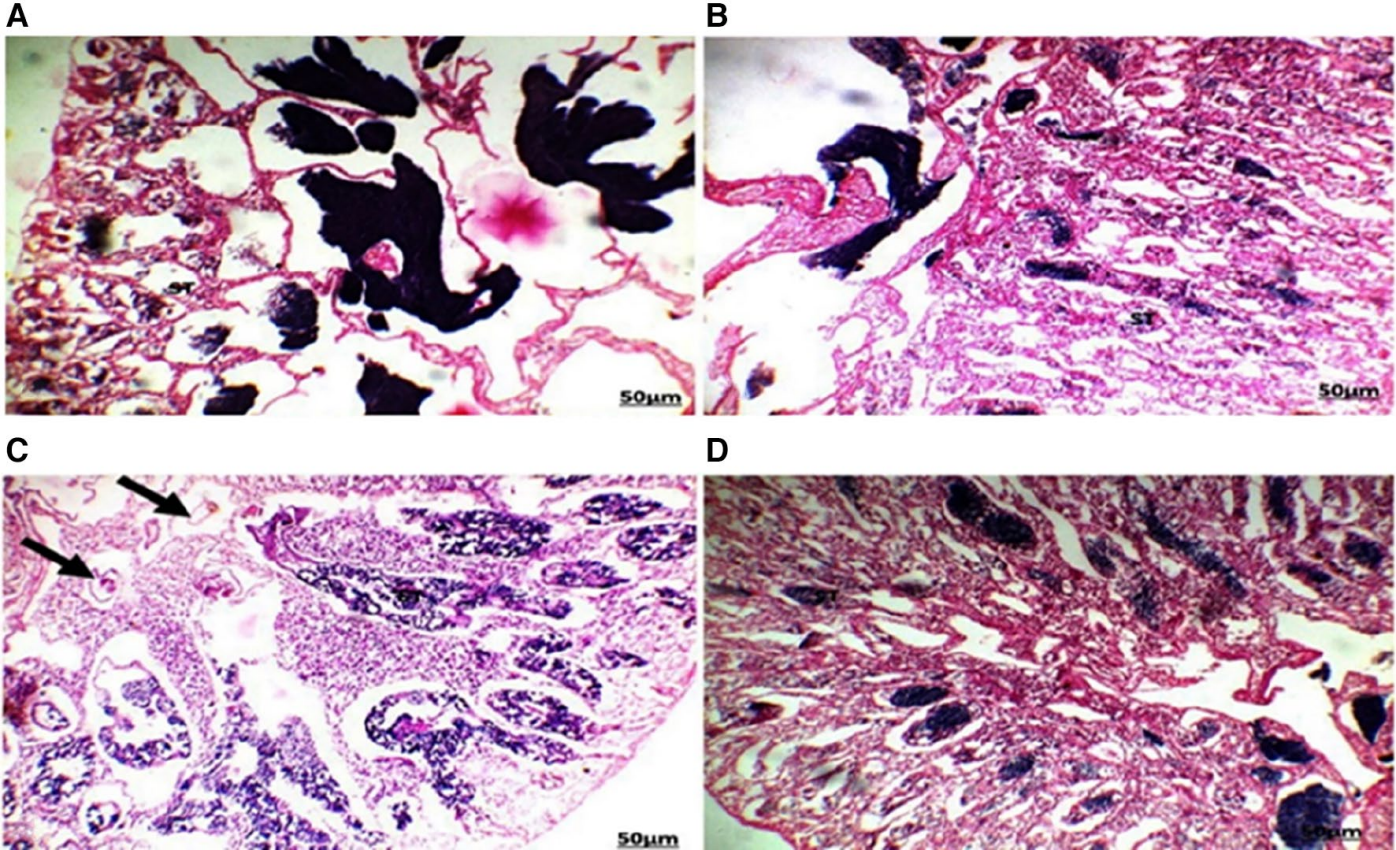
Section of Tilapia testis collected from area B during the summer (**A**), autumn (**B**), spring (**C**), and winter (**D**) seasons showed some empty degenerated testicular lobules with all the developmental spermatogenic stages and thick tunica albuginea. Spring season (**C**) showed encysted metacercaria (arrow) surrounded by necrotic tubules and inflammatory cells (HE, 100×)

The testes of fish collected from areas A and B during the four-season showed seminiferous tubules with different spermatogenic stages (Figs. 5 and 6), with the appearance of the spermatozoa in the lumen of some lobules. some lobules appear empty. A lot of seminiferous tubules with degenerated spermatogenic stages. Section of testis collected from area A during summer (Fig. 5A) showed small testicular lobules with thin tunica albuginea and only spermatogonia and appeared kidney in shape. Autumn (Fig. 5B) showed some testicular lobules with dense spermatozoa in the lumen and degenerated spermatogenesis. During spring (Fig. 5C) and winter (Fig. 5D) seasons showed some empty degenerated testicular lobules with all the developmental spermatogenic stages and thick tunica albuginea, the seminiferous tubules take the shape of parachute and broken. Section of testis collected from area B during summer, autumn, spring, and winter season (Fig. 6A–D) showed some empty degenerated testicular lobules with all the developmental spermatogenic stages and thick tunica albuginea. The Spring season (Fig. 6C) showed encysted metacercaria surrounded by necrotic tubules and inflammatory cells.

**Fig. 7:**
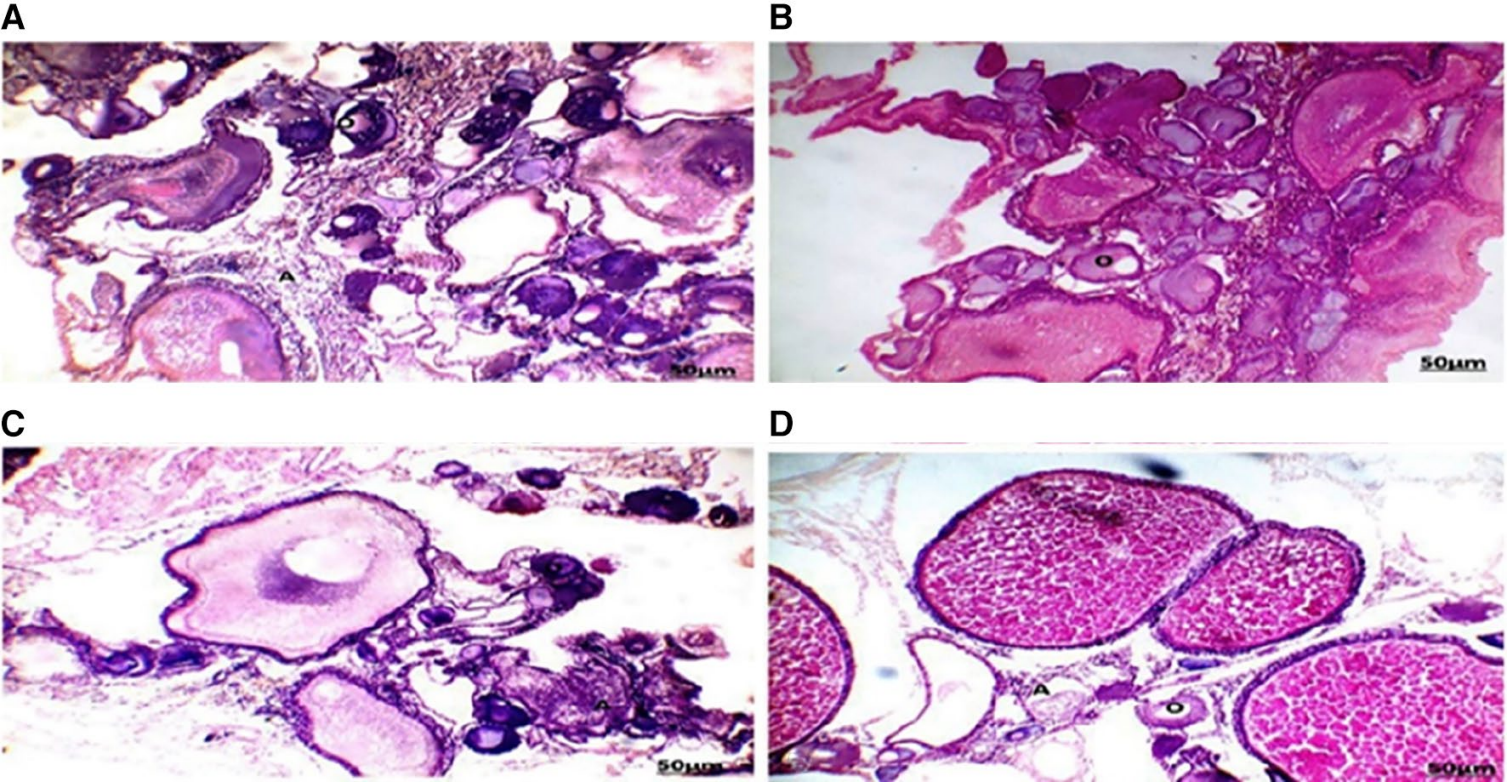
Section of tilapia ovary in area A during summer (**A**) showed yolk vesicles in the cytoplasm which appeared empty, the yolk vesicle appeared in the periphery of the cytoplasm. The oocyte is surrounded by zona radiata with a follicular epithelial layer (O). The autumn (**B**) showed the predominance of the perinuclear stage, the oocyte is polygonal with nucleoli in the periphery of the nucleus. The oocyte membrane is not differentiated (O). during the spring (**C**) showed yolk vesicles in the cytoplasm which appeared empty, and the yolk vesicle appeared in the periphery of the cytoplasm. The oocyte is surrounded by zona radiata with a follicular epithelial layer (O). During the winter (**D**) showed yolk globules in the periphery of the cytoplasm, the nucleus appeared granulated with irregular boundary, and the oocyte was surrounded by zona radiata and follicular epithelium (O) (HE, 100×)

**Fig. 8:**
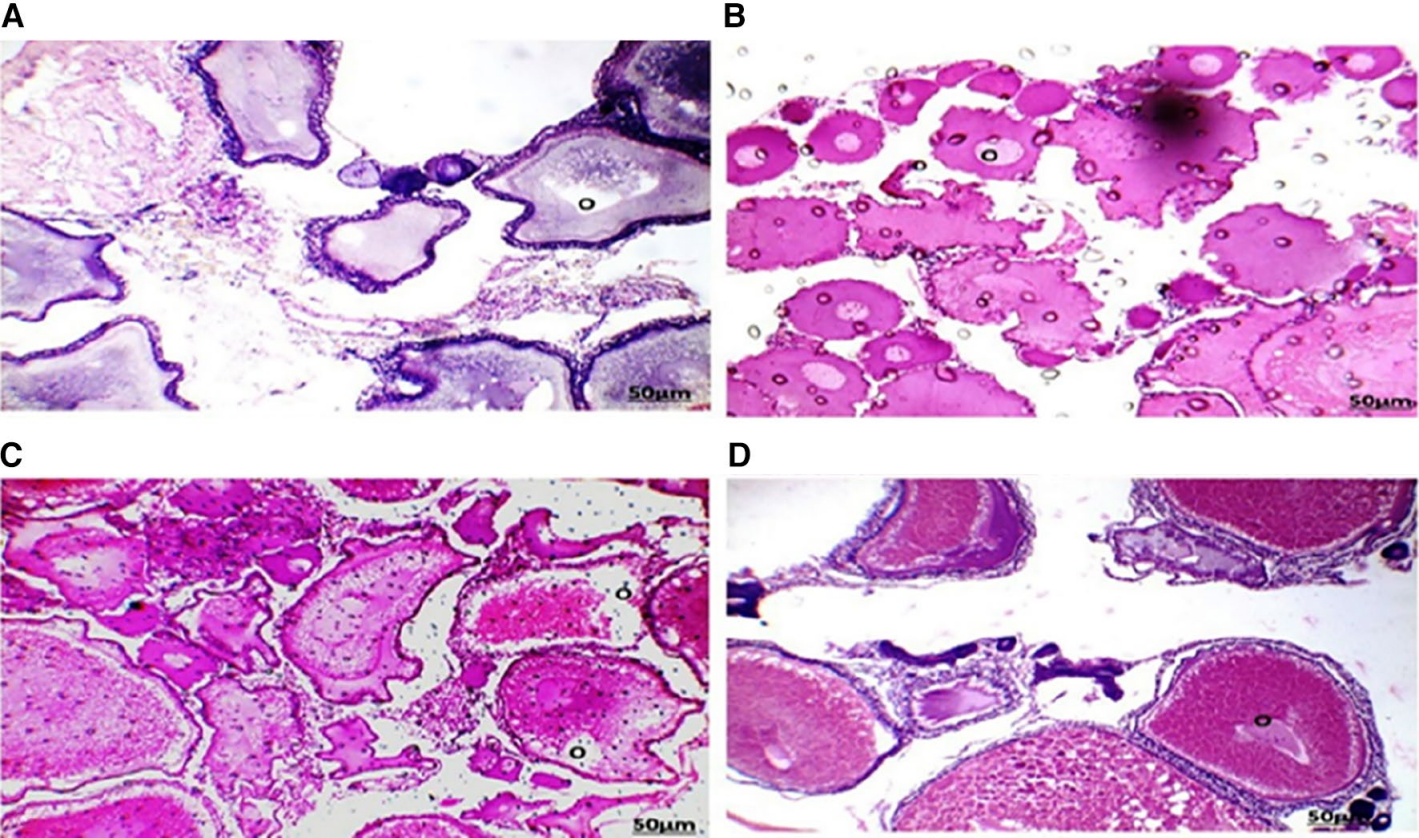
A section of the ovary in area B during summer (**A**) showed atretic oocytes lose their identity, the follicular epithelium thickened, and the nucleus dissolved in the oocyte material (O). During the autumn (**B**) showed atretic oocytes lose their identity, the follicular epithelium thickened, and the nucleus dissolved in the oocyte material (O). During spring (**C**) showed yolk globules in the periphery of the cytoplasm, the nucleus appeared granulated with irregular boundary, and the oocyte was surrounded by zona radiata and follicular epithelium (O). During the winter (**D**) showed a mature yolk stage, the absence of a nucleus, zona radiata became well-differentiated, and some oocytes its nuclei peripheral (O) (HE, 100×)

The ovaries of fish collected from areas A and B during the four-season showed a highly irregular structure with most of the oocytes being atretic (Figs. 7 and 8). Degeneration and necrosis of follicles were observed. Most of the follicles with hypertrophied theca. Section of the tilapia ovary in area A during the summer season showed yolk vesicles in the cytoplasm which appeared empty (Fig. 7A), the yolk vesicle appeared in the periphery of the cytoplasm. The oocyte is surrounded by zona radiata with a follicular epithelial layer. The autumn season showed the predominance of the perinuclear stage, the oocyte is polygonal with nucleoli in the periphery of the nucleus (Fig. 7B). The oocyte membrane is not differentiated. The spring season (Fig. 7C) showed yolk vesicles in the cytoplasm which appeared empty, and the yolk vesicle appeared in the periphery of the cytoplasm. The oocyte is surrounded by zona radiata with a follicular epithelial layer. The winter season showed yolk globules in the periphery of the cytoplasm, the nucleus appeared granulated with irregular boundaries, and the oocyte was surrounded by zona radiata and follicular epithelium (Fig. 7D).

Section of tilapia ovary in area B during the summer season (Fig. 8A) showed atretic oocytes lose their identity, the follicular epithelium thickened and the nucleus dissolved in the oocyte material. During the autumn season (Fig. 8B) showed atretic oocytes lose their identity, the follicular epithelium thickens, and the nucleus is dissolved in the oocyte material. The spring season showed yolk globules in the periphery of the cytoplasm, the nucleus appeared granulated with irregular boundary, and the oocyte was surrounded by zona radiata and follicular epithelium (Fig. 8C). Mature yolk stage, the absence of a nucleus, zona radiata became well-differentiated, some oocytes its nuclei peripheral were observed in winter (Fig. 8D).

## Discussion

This study examines certain biological aspects of *O. niloticus*, such as LWR and K. The LWR is a key factor that influences the growth of fish is the quality of the habitat of the fish (Jisr et al. 2018). To define the “condition” of an individual fish, the condition factor is determined using the connection between the weight and length of the fish (Nash et al. 2006). LWR was described by the equation: W = an L^b^ (Migiro et al. 2014). The value of the regression coefficient obtained was 0.71, 0.58, 0.93, and 0.83 at Muwies canal in summer, autumn, winter, and spring, respectively while R^2^ at San El-Hagar canal was 0.78, 0.86, 0.92, and 0.68 during summer, autumn, winter, and spring, respectively. The condition ratio was calculated using the equation, and there was a substantial link between length and weight: K = 100 (W/ L^3^) for *O. niloticus* was 1.76 and 1.79 (summer), 1.81 and 2.03 (autumn), 1.89 and 1.76 (winter) and 1.55 and 1.66 (spring) at Muwies and San El-Hagar canals, respectively which indicated good health condition during the study according to Khaironizam and Norma-Rashid (2002). The result of length-weight regression analysis in this work showed that all specimens in the Muwies canal exhibited allometric growth. The values of “b” exhibited negative allometric growth in the San El-Hagar canal. This means that *O. niloticus* became thinner or slender with an increase in its length as reported by Nehemia et al. (2012). *O. niloticus* showed positive allometric growth during winter (b = 3.29) and summer (b = 3.08) (positive allometric growth, fish become heavier and small specimens are in better condition than large ones) and exhibited negative growth during autumn and spring.

The current findings showed different values of the prevalence of parasites for different examined fish ranging from 50% for parasites in the San El-Hagar canal and 4.17% for parasites in the Muwies canal, seasonally. The prevalence of parasites was (10.42% and 35.42% in Muwies and San El-Hagar areas, respectively). The prevalence of parasites results lower than 34.2% recorded in the study of Hegazi et al. (2014) of Egyptian Lakes and that recorded in the investigation of Amaechi (2015) for prevalence (35.9%) of parasite infestation in *O. niloticus.* But, the prevalence of fish infection in Sharkia province was recorded in the study of Diab et al. (2006).

Variations in the prevalence of parasites might be attributed to differences in host immunity and the development of intermediate hosts (Gautam et al. 2018). The infection rate was higher in the wet season than in the dry season and this result agrees with Bichi and Dawaki (2010) who reported the same finding. Parasite prevalence was also assessed using different length categories (Amare et al. 2014). The parasite infection was shown to be greater in the fish with the largest size classes (> 20 cm) in the current investigation. This finding supports the findings of Locke et al. (2014), who found that the larger the fish, the greater the sensitivity to parasite infection because adult fish ingest a wide range of foods and feed in several ways. The incidence of parasite illnesses in fish species increases with their standard length, according to Sasal et al. (1999).

The physical and chemical properties of water showed that pH values for all the sampling locations ranged between 7.07 and 7.25. SO_4_^2−^ and Cl^−^ concentrations ranged between 0.15 and 0.9 mg/L and 0.10–1.60 mg/L, respectively. The concentrations of Na^+^, K^+^, Ca^2+^, and Mg^2+^ ranged between 0.4 and 0.90 mg/L, 0.1–0.3 mg/L, 1.20–1.75 mg/L and 0.75–1.55 mg/L, respectively. Mean levels (mg/L) of metals in water samples from Mueweis and San El-Hagar canal areas were 0.755 and 0.3175 (Fe), 0.0275 and 0.01 (Zn), 0.0505 and 0.12 (Mn), 0.0175 and 0.005 (Pb), and 0.01 (Cu), respectively, for each area (Mansour et al. 2019). Generally, levels of Fe and Pb in water from the two areas exceeded the World Health Organisation (WHO) stipulated limits of less than 0.3 for Fe and 0.010 for Pb, 3.0 for Zn, and 0.003 for Cd for drinking water, and levels of Cd recorded in Mueweis exceeded the stipulated WHO limits (Izah et al. 2016). Fe, Zn, Mn, Cu, and Pb were found in the liver and gonads tissue of *O. niloticus* in the Mueweis and San El-Hagar canal area (El-Shenawy et al. 2021a, b).

Moreover, the fish muscles contained levels of Fe, Zn, and Mn that were within the safe ranges established by the Codex Alimentarius Commission (Codex Alimentarius Commission 1995). As opposed to the threshold established by the Codex Alimentarius Commission, the levels of Pb in the muscles were up to 19.6 times higher. Based on the consumption habits of research participants, the hazard quotients (HQs) for Fe, Zn, and Mn for fish were less than 1, suggesting that these metals do not represent a risk to human health. Indicating that consumers face health concerns, HQs for Pb for tilapia in both research areas were larger than one. Because fishermen consume more fish, they are thought to be at higher health risk (El-Shenawy et al. 2021a).

The present study revealed no relationships were found between metal concentrations in the water of Muwies canal and with parasite abundance of tilapia fish collected from that area. Also, there were positive relationships between Fe or Zn, or Mn concentrations in water with parasite abundance in tilapia fish collected from the San Hagar canal. As the Fe, Zn, and Mn levels increase in the water, the parasite abundance increase. The difference in associations between the parasite abundance and the concentrations of metals in the two studied areas resulted from the difference in pollution sources (Mueweis canal area which received industrial pollution from factories and San El-Hagar area which received agricultural and domestic pollution).

Parasitic fish illnesses account for around 80% of all fish diseases, and they can have a severe impact on fish health by lowering weight increase, immunity, and death rates (Paperna 1991; Sitjà-Bobadilla 2008). The histopathological evaluation of fish target tissues is the endpoint in determining the danger of contaminants in the environment (Van der Oost et al. 2003). The influence of numerous anthropogenic pollutants on fish histopathological abnormalities can be utilized to determine the overall health of the fish. Pollutants can cause harmful effects on fish tissues before causing changes in the fish’s outward look and behavior (Kasumyan 2019).

The fish’s liver was responsible for food absorption, bile generation, detoxification, and body metabolic balance, which included carbohydrate, protein, lipid, and vitamin processing (Taddese et al. 2014). Stressors such as water contamination are known to disrupt the microcirculation of the hepatic parenchyma and caused a histopathological alteration to the liver (Abumourad et al. 2013). Poleksic et al. (2010) explained liver alterations in fish as a result of water pollution. The parasitic infected fish liver showed marked alteration in the histological structure. This was in line with the findings of Zhi et al. (2018), who observed an intense inflammatory response in fish after infection with the parasite. Hamouda and Bazh (2019) discovered lymphocyte infiltrations around parasite metacercaria, noting that the severity of the response was mostly determined by the cyst’s size and the infected tissue. According to Koca et al. (2008), tilapia fish liver infection revealed many cysts in the liver that are surrounded by a cellular inflammatory response. The pressure atrophy of the encysted metacercaria in liver cells, as well as the toxic waste products released by these cysts, led to severe hydropic degeneration of the hepatocytes in infected fish (Frasca Jr et al. 2018). The hypertrophy of the fish hepatocytes and bile duct observed in the present study might have occurred as a cellular stress response to an increased inflow of nutrients to liver cells and increased workload (Taddese et al. 2014).

Present observations of the histological study revealed that the testis of fish appeared abnormal in its architectures in the two studied areas with abnormal testicular lobule and spermatogenic stage. These findings agree with Ismail and Mahboub (2016).

The Nile Tilapia in this study had parasite infections that showed up in the testis tissues as a variable-sized encysted metacercarial infection surrounded by inflammatory cells. Pathological lesions of the testes coupled with inflammatory reactions were linked to a direct influence of the parasites on the organ’s wall, which resulted in cellular death compression and harmful inflammatory reactions (Walaa et al. 2019). In the present study, the histopathological alterations of ovary Tilapia collected from the two areas may be attributed to the effects of metals in the agricultural, industrial, and sewage wastes discharged into the two canals. Similar results were detected by Mazrouh and Mahmoud (2009) who showed a higher incidence of gonadal abnormalities of tilapia in a polluted area.

Based on the findings, it is possible to conclude that there is a significant link between biological research and Tilapia parasite abundance and the metals in which they live. It has been also shown that the tilapia inhabiting wastewater canals showed a marked deterioration in liver, testicular, and ovary histological structure. As can be seen from the above, water pollution harms fish health and reproduction in the examined locations, which is reflected in both economic development and human health.

This study provided important information for managing fishing ports and preventing the buildup of metals in water by demonstrating the state of the Mueweis canal and San El-Hagar’s metal pollution. These findings also point to the necessity of metals management and remediation, including the building of sewage treatment facilities, and the drafting of regulations for metals source reduction.

## Data Availability

Data supporting findings are presented within the manuscript.
